# How we diagnose Myelodysplastic syndromes

**DOI:** 10.3389/fonc.2024.1415101

**Published:** 2024-09-13

**Authors:** Howard S. Oster, Moshe Mittelman

**Affiliations:** ^1^ Department of Medicine, Tel Aviv Sourasky Medical Center, Tel Aviv University School of Medicine, Tel Aviv, Israel; ^2^ Department of Hematology, Tel Aviv Sourasky Medical Center, Tel Aviv University School of Medicine, Tel Aviv, Israel

**Keywords:** Myelodysplastic syndromes, cytogenetics, computer model, peripheral blood (PB), bone marrow examination, genetic mutations

## Abstract

The Myelodysplastic syndromes (MDS) are a heterogenous group of clonal bone marrow (BM) stem cell myeloid neoplasms, characterized by ineffective hematopoiesis that results in dysplasia in hematopoietic cells and peripheral cytopenias, especially anemia, and a propensity to leukemic transformation. The suspicion of MDS is raised by a typical but not specific clinical picture and routine laboratory findings, but the gold standard for MDS diagnosis is still BM examination with the presence of uni-or multi-lineage dysplasia and increased blast percentage, together with exclusion of other reasons. Cytogenetics is also an essential part of the diagnostic and prognostic processes. Flow cytometry and full genetic characterization are helpful but not mandatory for MDS diagnosis. This review summarizes the current steps of diagnostic approach for a patient suspected of having MDS. We also express our hopes that within the near future, non-invasive technologies, especially digital and peripheral blood genetics, will mature and be introduced into practice.

## Introduction

The Myelodysplastic syndromes (MDS) are a heterogenous group of clonal bone marrow (BM) stem cell myeloid neoplasms, characterized by ineffective hematopoiesis that results in dysplasia in hematopoietic cells and peripheral cytopenias, especially anemia, and a propensity to leukemic transformation (1–4). MDS incidence increases with aging and is approximately 5 cases per 100,000 people per year in the general population, on average, with a median age of onset of above 70 (3, 5, 6). Once patients are diagnosed with MDS, they are categorized using one of the classifications (7–11). Practically, most patients are assigned to the lower-(LR) or higher-risk (HR) groups. These classifications assist in diagnosis but serve mainly as a prognostic tool and to direct management.

Here we focus on the diagnosis of MDS, both the classic, standard approach as well as some modern modalities which are being tested as diagnostic tools.

Unfortunately, since we are dealing with a heterogenous group of disorders, in contrast with many other diseases, there is no single specific diagnostic test or definitive diagnostic criteria for MDS. The diagnostic process is based on various clinical and laboratory features with exclusion of other diseases.

## When to suspect MDS

The suspicion is raised by the clinical and laboratory picture in the elderly. Clinically, MDS symptoms are non-specific. Patients may be asymptomatic, or may have non-specific complaints such as weakness and fatigue. They may also have cardiac complaints, due to the common anemia (**Table 1A**) (1, 3, 5, 12, 13). Decreased neutrophil count might be associated with recurrent infections and patients might have epistaxis, gingival bleeding or easy bruising if their platelets are low in number or dysfunctional (14).

**Table 1:** Making the diagnosis of MDS.

	Finding	Comments
**1A: History (suggestive) (** 1, 3, 5, 12, 13, 15–17)	Weakness and fatigue	Associated with anemia
	Shortness of breath/angina	Associated with anemia
	Recurrent infections	Associated with neutropenia
	Bruising, bleeding	Associated with thrombocytopenia
	Family history of BM disease	
	History of chemo/radiation therapy	
**1B: Physical exam (suggestive)**	Pallor	Associated with anemia
	Splenomegaly	In CMML
**1C: Routine laboratory abnormalities (** 1, 7, 9, 13, 17–19)	Anemia	
	MCV elevation	
	RDW widening	
	Leukopenia	
	Neutropenia	
	Thrombocytopenia	
	Monocytosis	
	Elevated CRP/ESR	
	Normal chemistry	Unless comorbidity
**1D: Bone Marrow, mandatory (** 3, 6, 7, 9, 10, 15)	Cellularity (general) Hypo, hyper, normal	Typical, but not diagnostic
	Cell number in each lineage	Helpful, but not diagnostic
	Dysplasia in any line	Mandatory for diagnosis
	Blast percentage	For diagnosis and prognosis
	Ringed sideroblasts	In sideroblastic anemia
	Monocytosis	In CMML
	Cytogenetics	For diagnosis and prognosis
**1E: Bone Marrow, recommended (** 3, 6, 12, 23, 24, 30, 42)	Flow cytometry	Helpful
	Genetics, somatic	Increasingly used
	Genetics, germline	Where familial disease is suspected

When MDS is suspected, a careful history must be taken to search for other causes of anemia, including nutritional (folic acid, iron and vitamin B12, especially in vegetarians), medications, alcohol, tobacco, or viral infection. The patient’s history can also determine if there had been exposure to radiation or chemotherapy, or if there is a familial predisposition to bone marrow disease (15, 16).

Other BM diseases, e.g. paroxysmal nocturnal hemoglobinuria, aplastic anemia and myeloproliferative neoplasm (MPN), must also be excluded by history (3, 15, 16).

Physical examination (**Table 1B**) is often unremarkable or non-specific, with no abnormal findings or only pallor. Bruising, and other bleeding evidence may be found in thrombocytopenic patients. Hepatomegaly and mainly splenomegaly is common in the MDS chronic myelomonocytic leukemia (CMML) subtype (1, 3, 7).

Laboratory findings: MDS laboratory findings are not specific (**Table 1C**). Erythrocyte sedimentation rate (ESR) and C-reactive protein (CRP) are often elevated (17). More than 90% of MDS patients will have anemia; approximately 50% of these patients will be anemic with hemoglogin (Hb) less than 10g/dl (9, 13). The anemia is typically macrocytic with a mean corpuscular volume (MCV) that tends to be high or high normal (1, 3, 7), although not as high as in B12 deficiency. Red cell distribution width (RDW) may be widened, especially in more severe disease (18, 19). Patients with MDS tend to lack the increase in reticulocyte count in contrast with hemolytic anemia (15). About 50% of MDS patients are pancytopenic. Leukopenia and neutropenia with absolute neutrophil count (ANC) less than 800x10^9^/L is found in 18% of patients and low platelet count (< 100x10^9^/L) is observed in 40% of MDS patients (9, 13). Platelet function may be defective as well (14). Monocytosis is the hallmark of CMML, but the recent 2022 classifications lowered the threshold to 0.5x10^9^/L (10, 11). This now includes what used to be referred to as oligomonocytic CMML.

Typically, routine blood chemistry is expected to be normal in MDS, unless there is a comorbidity associated with anemia. Serum iron and iron saturation as well as serum ferritin might be elevated in the MDS sideroblastic subtype.

Since MDS diagnosis is established by exclusion, blood chemistry should exclude nutritional deficiencies, like iron and especially folic acid and vitamin B12, both can cause macrocytic anemia. Blood chemistries should rule out underlying liver or kidney disease, and serology for hepatitis viruses B and C, and CMV, as well as HIV and parvovirus B19 should also be performed.

The peripheral blood (PB) smear might be typical and helpful, but not specific or diagnostic. The red blood cells (RBC) tend to demonstrate anisocytosis or poikylocytosis (7). Occasionally nucleated RBC are observed. The white blood cells (WBC) can show an increased number of immature myeloid cells (“left shift”) with hypolobulation (“Pelger”-like cells) and hypogranulation. PB platelets might be distorted, clumped, large (megaplatelet), or the number might be low. Persistent monocytosis would suggest CMML (7, 15), assuming that other etiologies for monocytosis have been excluded. Often, the PB smear helps to diagnose another hematologic disease and rule out MDS. For example, thrombocytosis or leukocytosis, would suggest an MPN, or at least an overlap MDS/MPN (see below).

Imaging is not a part of the MDS diagnostic process, unless hepatosplenomegaly is evaluated.

In summary, the combination of symptoms and laboratory findings with exclusion of other reasons for anemia or pancytopenia, might raise the suspicion of MDS (**Tables 1A–C**), however, none of these findings or even several of them, are enough to establish MDS diagnosis.

## Bone marrow examination – the gold standard for MDS diagnosis

Once MDS is suspected, other causes of anemia or cytopenia are ruled out, and the patient is defined as having an unexplained anemia (or cytopenia), the next and definitive step in the diagnostic workup is a bone marrow (BM) evaluation, still the gold standard for the diagnosis of MDS (**Table 1D**). As the organ of blood cell production, BM examination is used to assess abnormalities of blood cells and their hematopoietic precursors, reflecting MDS pathogenesis and establishing the diagnosis. A full BM examination consists of both an aspirate and a biopsy. The main component to establish MDS diagnosis is morphology. Often additional tests are performed, including special staining, cytogenetics, immunophenotyping and more recently, genetics.

BM Morphology: Using May-Grunwald-Giemsa staining, BM aspirate is essential to assess the morphology of single cells. BM cellularity can be estimated but it is not accurate enough, in contrast with that of the biopsy. Typically, the aspirate is characterized by abnormal BM cellularity (hypocellular or hypercellular), but mainly by dysplasia, which can be found in any cell lineage. Dysplasia is considered significant if more than 10% of the nucleated cells of a given line have such changes (3). In the erythroid compartment, the dyserythropoietic changes may include megaloblastosis (similar to B12 deficiency), binuclearity or fractured nuclei (karyorhexis), irregular nuclear edges or internuclear bridging, cytoplasmic inclusions or bridging, vacuolization, fringed cytoplasm or incomplete hemoglobinization (7, 20, 21). The myeloid line may show an increased number of young immature cells, anisocytosis or changes in the shape of the nucleus, including hypolobulation or hypersegmentation. There may also be pseudo granules or cytoplasmic hypogranulation/degranulation. We specifically search for blasts (myeloid in MDS), which are identified by their high nuclear/cytoplasmic ratio, nucleoli, fine nuclear chromatin and cyoplasmic basophilia. Blasts may have granules or Auer rods. Blasts are counted and reported as percentage of nucleated BM cells. The megakaryocytic lineage can have large monolobular forms, small binucleated elements, dispersed nuclei, micromegakaryocytes and degranulation (3, 7). The smears are also stained for iron (Prussian blue) to assess for the presence of ringed sideroblasts (RS). The presence of RS > 15% of BM nucleated RBC diagnoses the MDS subtype of refractory anemia with ringed sideroblasts (RARS) (7). BM aspirate also serves for special tests, especially for MDS exclusion and to establish other hematologic disorders.

The BM trephine biopsy is important for evaluating the BM cells in their milieu, and provides information on cellularity, although this parameter has not been found to be critical for MDS diagnosis or prognosis (3, 7, 22). BM biopsy might also identify possible fibrosis. BM biopsy is less reliable in evaluating the morphology of single cells or counting blasts. Stains include hematoxylin/eosin as well as Giemsa. Specimens also undergo immunohistochemistry stains for Glycophorin A or C for the erythroid line, CD34 and CD117 for blasts, CD61 or CD42b for the megakaryocytes, KP1/CD68 or PGM1/CD68R for monocytes, CD20 for the B-cell line, and CD3 for the T-cell line (3). Reticulin stain or Gomori’s silver impregnation are used to evaluate for BM fibrosis (3, 15). Occasionally, other cells can be identified that may lead to other diagnoses like cellular metastases from other malignancies.

In summary, BM examination, especially the dysplastic features and blast percentage are mandatory in establishing MDS diagnosis (**Table 1D**). Moreover, once diagnosed, these BM findings, and especially the blast percentage further assist in categorizing and predicting the prognosis of the patient, according to the various classifications (4, 7, 8, 10). Finally, the blast % also distinguishes between HR-MDS and acute leukemia, although the line between these two entities declines over the years from 30% to 10% (depending on genetic signatures) (4, 7, 10, 11).

Flow cytometry: Immunophenotyping by flow cytometry (FC) is an adjunct diagnostic tool assisting in establishing MDS diagnosis and might also serve for follow up (**Table 1E**) (3, 12). The technology is based on detection of multiple aberrancies on a particular cell, as opposed to any single marker. The combined profile of several markers, can distinguish MDS from other cytopenias (23, 24). For example, a score that identifies at least two of the following criteria – increased CD34+ progenitors in nucleated BM cells, a decrease in B cell progenitors among CD34+ cells, change in CD45 expression on myeloid progenitor cells as compared with lymphocytes, and a decrease in sideward light scatter (SCC) of neutrophils compared with lymphocytes – demonstrated a specificity of over 90% in patients with low risk MDS (25, 26). Other markers can increase sensitivity and even identify cellular dysplasia. Unfortunately, due to high cost and the need for special facilities, this method has not been widely adopted globally, and is regarded as rather helpful but not mandatory.

Cytogenetics: Cytogenetics is performed with a combination of G-banding and FISH techniques. While it may not be required to establish MDS diagnosis, no diagnostic workup is complete without performing it (3, 6, 9). (**Table 1D**). At least 20 cells in metaphase should be examined. Thus, applying cytogenetics with the typical chromosomal abnormalities assists in the diagnosis. The common MDS cytogenetic findings are chromosomes 5 (deletion or monosomy), chromosome 7, + 8 (27). Cytotogenetics is even more important in predicting prognosis (8, 9). In the WHO 2016 classification of MDS, the use of cytogenetics was important for diagnosis especially where the existence of dysplasia is not seen at all, is less than 10% in all cell lineages, or is equivocal. Such patients were then seen as MDS-unclassifiable (4). In the current classification systems, that has been replaced by incorporation of clonal cytopenia of undetermined significance (CCUS) (10, 11), but the principle is the same.

Genetics: Over the last couple of decades, it has become clear that like other malignancies, genetic mutations are responsible for the development of the malignant clone(s) in MDS and these genetic signatures control the disease course (**Table 1E**) (28). We know today that 90% of MDS patients do harbor myeloid mutations (3, 29–31), (**Table 2**) with 2-3 mutations per patient at MDS diagnosis, on the average. Many mutations were described in MDS, but six are found in at least 10% of MDS patients: SF3B1, TET2, SRSF2, ASXL1, DNMT3A and RUNX1 (6, 31–41). However, in contrast with other hematologic neoplasms, CML or CLL for example, introduction of genetics into clinical practice, both for diagnosis and prognosis (42) is still in its infancy. Several tough hurdles still prevent broad genetic application (6, 30, 31, 43–46). We learned that not all mutation were born equal - there are driver mutations, obviously more important clinically while other are just passengers. Often there are not only founding driver mutations, but also sub-clonal driver mutations which generate a new clone with both the newly acquired as well as the original founding mutations. Certain mutations are associated with favorable (isolated SF3B1 (32, 33)) or poor (TP53 (31, 47), RUNX1(40, 41)) prognosis. The variant allele frequency (VAF) and hotspot of the mutation appear to be important. The function of mutations as well as occurrence of co-mutations and gene-gene interaction is still not elucidated. We have also learned that mono-allelic mutation, for example TP53, is associated with a better prognostic phenotype as opposed to bi-allelic mutation (48). Finally, no mutation has been found to be unique or diagnostic for MDS, with the only exception of SF3B1 mutation which is recognized today as an MDS defining signature (10, 11, 15). Moreover, these mutations have been found in healthy aging people too, and most of them will never become sick, the phenomenon defined as age-related clonal hematopoiesis (ARCH) (49, 50), or clonal hematopoiesis of indeterminate potential (CHIP) (51, 52). **Table 2** lists other mutations (with references) that have an association with MDS.

**Table 2:** Genetic mutations in MDS.

Gene	Reference
*SOMATIC MUTATIONS*	
*SF3B1*	Yoshida et al. (32), Malcovati et al. (33)
*TET2*	Delhommeau et al. (34), Langemeijer et al. (35)
*RUNX1*	Chen et al. (40), Dicker et al. (41)
*ASXL1*	Bejar et al. (31), Thol et al. (37)
*SRSF2*	Yoshida et al. (32), Thol et al. (36)
*TP53*	Bejar et al. (31), Padua et al. (47)
*U2AF1*	Yoshida et al. (32), Graubert et al. (86)
*NRAS/KRAS*	Dicker et al. (41), Paquette et al. (87)
*DNMT3A*	Walter et al. (39), Thol et al. (38)
*ZRSR2*	Yoshida et al. (32), Thol et al. (36)
*EZH2*	Nikoloski et al. (88), Ernst et al. (89)
*IDH1, IDH2*	Bejar et al. (31), Kosmider et al. (90)
*ETV6*	Bejar et al. (31)
*CBL*	Bejar et al. (31), Makishima et al. (91)
*NPM1*	Bejar et al. (31), Dicker et al. (41)
*JAK2*	Bejar et al. (31), Steensma et al. (92)
*SETBP1*	Piazza et al. (93), Makishima et al. (94)
*SF3A1*	Yoshida et al. (32)
*SF1*	Yoshida et al. (32)
*U2AF65*	Yoshida et al. (32)
*PRPF40B*	Yoshida et al. (32)
*GERMLINE MUTATIONS*	
*DDX41*	Cazzola et al. (53), Homan et al. (62), Li et al. (54), Makishima et al. (55, 60), Kennedy et al. (56), Bannon et al. (57), Hamilton et al. (58)
*GATA2*	Cazzola et al. (53), Calvo et al. (63), Homan et al. (62), Largeaud et al. (65), Robbins et al. (64), Kennedy et al. (56), Bannon et al. (57), Locatelli et al. (66)
*RUNX1*	Cazzola et al. (53), Homan et al. (62, 67), Bannon et al. (57)
*ANKRD26*	Cazzola et al. (53), Homan et al. (67), Bannon et al. (57)
*ETV6*	Cazzola et al. (53), Homan et al. (67), Bannon et al. (57), Locatelli et al. (66)
*SAMD9/SAMD9L*	Kennedy et al. (56), Locatelli et al. (66), Sahoo et al. (68), Nagata et al. (69)
*SRP72*	Bannon et al. (57), Kirwan et al. (70), Locatelli et al. (66)
*BLM*	Kim et al. (71)
*BRCA1, BRCA2*	Kim et al. (71), Hamilton et al. (58)
*CTC1*	Kim et al. (71)
*ERCC4*	Kim et al. (71)
*ERCC6*	Kim et al. (71)
*FANCI, FANCM*	Kim et al. (71)
*PALB2*	Kim et al. (71)
*SBDS*	Kim et al. (71)

Modified and updated based on Malcovati et al., 2013 (3)

A relatively new area is the germline mutation in MDS. Until several years ago we looked at germline mutations as a pediatric problem. Over the last couple of decades several such mutations are not only observed but also result in a clinical phenotype, which might be detected only at an adult age. Examples are DDX41 (53–62), GATA-2 (53, 56, 57, 62–66), and others (53, 56–58, 66–71) (**Table 2**).

The challenges we face now is how to detect these individuals, how to follow and manage them, and most importantly who of the family members to screen. We expect to have some of the answers within the next few years.

In summary, one cannot underestimate the role of genetics in diagnosis, as well as in the pathogenesis and prognosis (31, 42), but in 2024 we are still in the beginning of this era, and the genetic profile, although routinely performed in many parts of the world, is still not a mandatory tool in the diagnostic workup. Of course, the high cost and lack of professional skills to perform this analysis further prevents its wide application.

It is noted that some of these mutations found (not only but also) in MDS already serve as targets for treatment. Examples are the APR-246 targeting TP53 mutations (72) and the IDH1/2 inhibitors (73).

## MDS/MPN overlap syndrome

When a suspected patient undergoes a workup diagnostic procedure, one has to consider both disorders with overlapping features, as well as diseases that should be excluded. Patients with myeloproliferative neoplasms (MPN) share several clinical, laboratory and imaging features with MDS. Both the WHO and the ICC guidelines devote attention to such patients (10, 11, 74). The debate whether the MDS subgroup CMML is indeed MDS or should be re-classified with MPN has been with us since the first classification (7). MDS/MPN overlap is suggested when there is splenomegaly and/or elevated WBC (>13k) with or without monocytosis, now > 0.5x10^9^/L (CMML) and/or PLT count (>450k). It can also be associated with ring sideroblasts (with SF3B1) (11, 15).

## Pre-MDS states

Several pieces of evidence suggest that MDS develops over time (75) in which the malignant clone evolves before the clinical disease is diagnosed. The occurrence of myeloid mutations in healthy individuals with a higher tendency to further evolve into full blown myeloid diseases, especially MDS, further supports this concept (49, 50). Like other hematologic neoplasms such as multiple myeloma (monoclonal gammopathy of undetermined significance) and chronic lymphocytic leukemia (monoclonal B-cell lymphocytosis), pre-MDS states are recognized too. These entities include idiopathic cytopenia of undetermined/unknown significance (ICUS), many of which end up being clonal cytopenia of unknown significance (CCUS). ICUS is characterized by cytopenia without a known cause and without the minimal criteria to establish MDS diagnosis (76–78). In CCUS, a clonal myeloid mutation is observed, with some overlap with ARCH and CHIP (51), however, it cannot be (still) defined as MDS. There may also be dysplasia without cytopenia (IDUS, idiopathic dysplasia of unknown significance) (76, 79), and BM clonal changes without cytopenia. Does it make sense to diagnose these pre-MDS states? Probably yes. Although no current therapeutic policy justifies intervention in these disease states, one can foresee such in the future, especially considering the coming biological less-invasive technologies. How to diagnose these conditions? It is likely that genetic studies and identification of individuals at risk (e.g. germline mutations) might assist. However, one cannot ignore the social, ethical and financial considerations making it still very difficult at present.

## Novel approaches to diagnose MDS

As stated above BM examination and its morphology is still the gold standard tool to diagnose MDS. Many still believe that the information obtained, including the morphological findings and the blast percentage cannot be replaced by any other method. However, since this examination is invasive and painful, and morphological evaluation is somewhat subjective with high interobserver variations (80), one would want to avoid it. Work toward this goal has progressed along two lines and is still under investigation but quite promising.

The first approach applies digital tools comparing numerous data collected from large numbers of patients, to data obtained from healthy subjects. Our group, in collaboration with the European MDS group, analyzed such data collected from 501 MDS patients and compared them to 501 controls. We developed a simple diagnostic model applying 10 simple parameters that are easily available (age, sex, hemoglobin, white blood cells, platelets, mean corpuscular volume, neutrophils, monocytes, glucose, and creatinine) (81, 82). These variables of a suspected individual can be applied and the model was designed to provide one of three possible predictive conclusions: probably MDS (pMDS), probably not MDS (pnMDS) and indeterminate. We found that we could predict or rule out MDS in over 80% of patients with unexplained anemia with an area under the receiver operator characteristics (ROC) curve (AUC) of 0.96. We recently validated the model using data from patients and controls who had not been included in the development of the model (83). Also, external validation was performed by the Dusseldorf group, using data from a different center and found the model especially useful in ruling out MDS (84).

The second approach to skip BM examination in the diagnostic workup is based on the assumption that most relevant information, especially genetics, can be found in the PB if we have the right methodology to use it. One example is the work recently presented, identifying PB CD34+ hematopoietic stem and progenitor cells (HSPC) and performing single cell RNAseq, which can potentially diagnose MDS or pre-MDS states (85). These approaches are still investigational and not the standard, however, it is likely that such non-invasive methods will obviate the need for BM evaluations in many patients for diagnosing MDS.

## Summary

In 2024 we are still conservative regarding the diagnosis of MDS. To make the diagnosis of MDS, some tests are mandatory, especially BM examination (aspirate and/or biopsy) identifying dysplasia in one or more and enumeration of blasts, as well as exclusion of other reasons for anemia (or cytopenia). Cytogenetics is also an essential part of the diagnostic process. Suspicious clinical picture, macroctyic anemia (or cytopenia), peripheral blood abnormalities, presence of BM ringed sideroblasts, flow cytometry and myeloid somatic mutations as well as other genetic assays are helpful and recommended but not critical for MDS diagnosis. We express our hopes that within the near future, non-invasive technologies, such as those described (digital and PB genetics) or others, will mature and be introduced into practice.
